# Enhanced Fluoride Removal Performance from Water by Calcined-State Mayenite (Ca_12_Al_14_O_33_): Adsorption Characteristics and Mechanism

**DOI:** 10.3390/ma18102189

**Published:** 2025-05-09

**Authors:** Wenyun Zhu, Zhonglin Li, Yonghang Tan, Guixiang He, Xuexian Jiang, Yibing Li, Weiguang Zhang, Xiaolan Chen

**Affiliations:** 1 Department of Materials Science and Engineering, Guilin University of Technology, Guilin 541004, China; zwy970316@163.com (W.Z.); dahe121133@163.com (Z.L.); 18877071982@163.com (Y.T.); hgx@glut.edu.cn (G.H.); zhangwg@glut.edu.cn (W.Z.); 2 Collaborative Innovation Center for Exploration of Nonferrous Metal Deposits and Efficient Utilization of Resources, Guilin University of Technology, Guilin 541004, China; 3 Department of Metallurgical and Resources Engineering, Guilin University of Technology at Nanning, Nanning 530001, China; 15207814314@163.com

**Keywords:** mayenite, calcination, fluoride removal, wastewater treatment, adsorption mechanism

## Abstract

This study achieved the preparation of budget-friendly stratified Ca-Al adsorbents using a simplified precipitation synthesis route with subsequent pyroprocessing, showing superior defluoridation capabilities in aqueous environments. The structural properties and defluoridation performance of the adsorbents were systematically investigated by optimizing critical synthesis parameters, including calcium-to-aluminum molar ratios, the solution pH during co-precipitation, and calcination temperature. Characterization results revealed that the optimal sample (prepared at a Ca/Al ratio of 2:3, initial pH of 10, and calcination temperature of 600 °C) exhibited a high specific surface area, ordered mesoporous structure, and abundant surface hydroxyl groups, facilitating efficient fluoride adsorption. Batch adsorption experiments demonstrated significant effects of adsorbent mass, solution pH, and initial fluoride concentration on removal efficiency. The isothermal adsorption characteristics conformed to the Langmuir model, complemented by pseudo-second-order kinetic compliance, which jointly confirmed chemisorption-dominated monolayer coverage. Notably, the maximum adsorption capacity reached 263.33 mg g^−1^, surpassing most comparable adsorbents reported in the literature. The material maintained a superior fluoride removal performance across a wide pH range (4~12) and exhibited superior recyclability. Rapid adsorption kinetics were observed, with equilibrium achieved within 60 min. The material showed a good removal effect in actual fluoride-containing smelting wastewater, which further proved its application potential. In addition, the analysis of the adsorption mechanism showed that the removal of fluoride was mainly achieved through the coordination between fluoride and metal ions and the ion-exchange reaction with surface hydroxyl groups. These findings suggest that the adsorbent has significant prospects for practical water quality fluoride removal applications.

## 1. Introduction

As a common environmental pollutant, fluoride is widely found in natural water bodies and industrial wastewater [1]. Excessive concentrations of fluoride in water not only pose a threat to human health but may also lead to diseases such as skeletal fluoride deposition, abnormal bone development, and dental fluorosis and, in severe cases, even affect the structure and function of bones. In addition, an excessive accumulation of fluoride can also upset the balance of aquatic ecosystems and cause ecological hazards [2,3]. According to the standards of the World Health Organization (WHO), the concentration of fluorine in drinking water should be controlled at less than 1.5 mg L^−1^ [4]. However, in some areas, due to natural geological effects or industrial pollution, the concentration of fluorine in water bodies far exceeds this standard, resulting in fluorine pollution becoming a major environmental problem that needs to be solved urgently. Therefore, the development of efficient and economical water treatment technologies to remove fluoride ions from water has become an important research direction in the field of water treatment [5].

Currently, common fluoride removal methods include adsorption [6,7] reverse osmosis [8,9], ion exchange [10,11], and precipitation [12,13]. Although effective, reverse osmosis is difficult to apply on a large scale due to its high cost and membrane clogging [14]. Ion exchange requires expensive resin materials and is susceptible to interference from competing anions, and it requires frequent replacement or regeneration [15]. The precipitation method is less costly, but its efficiency is limited by water quality conditions and may trigger secondary pollution [16]. In contrast, the adsorption method has become a more ideal method for defluoridation because of its easy operation, high cost-effectiveness, good treatment effect, and low impact on the environment [17,18,19]. However, the commonly used adsorbents, such as activated carbon, alumina and its base adsorbents, mixed metal oxides, and clay materials, generally have a low adsorption capacity, poor pH adaptability, and secondary pollution, which limits their wide application [20,21].

In recent years, layered double hydroxides (LDHs), also known as anionic clays, have shown excellent performance in various fields such as selective adsorption, catalysis, and separation, due to their unique interlayer structure and large specific surface area [22]. The anions in the interlayers of LDHs are removed during a high-temperature calcination process to form layered oxides (LDOs). The general formula of LDHs can be expressed as ${{[M}_{1–X}^{2+}M_{X}^{3+}{\left(\mathrm{OH}\right)}_{2}]}^{2+}{[A}_{X/m}^{m-}\cdot nH_{2}O]$, where M^2+^ and M^3+^ are divalent and trivalent, respectively; 3+ are divalent and trivalent metal ions, respectively; and A is an interlayer anion [23]. Due to their unique molecular structure, the charge density and interlayer spacing can be precisely adjusted by regulating the ratio of divalent metal (e.g., Ca^2+^, Mg^2+^) and trivalent metal (e.g., Al^3+^, Fe^3+^) ions, which endows LDO materials with a high specific surface area, a strong anion-exchange capacity, and selective adsorption properties for anionic pollutants [24]. Therefore, LDOs are considered ideal adsorbents, especially for oxygen anions such as fluorine [25]. However, the existing LDO materials still suffer from several limitations, such as slow adsorption kinetics, a limited pH tolerance (optimal pH range of 5–7), and irreversible collapse of the structure during regeneration. Thus, the development of LDO materials with a high adsorption capacity, wide pH adaptability, and good regeneration properties remains an important challenge in this field [26,27].

Based on the structural properties of layered bimetallic oxides (LDOs), this study innovatively developed a high-efficiency fluoride removal adsorbent, baked-state calcium aluminate (Ca_12_Al_14_O_33_). Using an optimized one-step co-precipitation method and a precisely controlled temperature calcination process, an active material with abundant surface hydroxyl groups and an ordered mesoporous structure was successfully synthesized. Its maximum fluoride adsorption capacity reached 263.33 mg g^−1^, which is significantly improved compared with most existing similar materials. Moreover, the synergistic fluoride removal mechanism, involving metal ion coordination and surface hydroxyl group ion exchange across a wide pH range (4~12), was systematically elucidated, overcoming the pH sensitivity limitations of traditional adsorbents. Through an integrated Na_2_CO_3_ elution–thermal regeneration cycling process, the material maintained 65.9% of its adsorption efficiency after five cycles, which is significantly better than the regeneration performance of current materials. The goal of this study is to develop a low-cost, high-capacity, and regenerable adsorbent material, offering a novel, efficient, and cost-effective solution for fluoride pollution control.

## 2. Experiment Section

### 2.1. Materials

Aluminum nitrate (Al(NO_3_)_3_·9H_2_O), calcium nitrate (Ca(NO_3_)_2_·4H_2_O), sodium hydroxide (NaOH), nitric acid (HNO_3_), sodium fluoride (NaF), anhydrous ethanol (CH_3_OH), acetic acid (CH_3_COOH), sodium chloride (NaNO_3_), trisodium citrate (C_6_H_5_Na_3_O_7_·2H_2_O) and all reagents mentioned in the text were of analytically pure grade with purity greater than 99% without further purification, were purchased from Xilong Science Co. Ltd. (Shantou, Guangdong, China), deionised water was homemade by the laboratory. A standard stock solution of 1000 mg L^−1^ fluoride was prepared by dissolving 2.21 g NaF in 1000 mL of deionized water at room temperature. Various experimental solutions were prepared by diluting the stock solution appropriately [28].

The actual fluorine-containing wastewater used in the experiment came from Guangxi Southland Copper Co., Ltd. (Chongzuo, China). The concentration of F was about 218.29 mg L^−1^, and the pH was 4.7. The composition of the actual F-containing smelting wastewater is shown in Table 1.

### 2.2. Preparation

A batch of calcined-state mayenite (Ca_12_Al_14_O_33_) adsorbent materials were prepared by a simple co-precipitation method and calcination process. In a typical synthesis process, Ca(NO_3_)_2_·4H_2_O and Al(NO_3_)_3_·9H_2_O were dissolved in 120 mL of 40% ethanol in molar ratios of 1:4, 2:3, 1:1, 3:2, and 4:1, respectively, with ethanol acting as a dispersant. Then, under strong magnetic stirring, 5 mol L^−1^ of NaOH solution was slowly added dropwise, and the pH of the mixed solution was adjusted to 10 with continuous stirring for 12 h. At the end of the reaction, the solid precipitate was collected by centrifugation and washed several times with deionized water and ethanol and dried at 80 °C for 12 h. The samples were numbered CA14-10, CA23-10, CA11-10, CA32-10, and CA41-10. In order to optimize the best synthesis conditions, adsorbents with a molar ratio of Ca to Al of 2:3 were prepared at a solution pH 7, 8, 9, and 11, noted as CA23-7, CA23-8, CA23-9, and CA23-11, respectively. In addition, the CA23-10 samples were calcined at 300, 400, 500, 600, and 700 °C for 2 h with a temperature increase rate of 5 °C·min^−1^, denoted as CA23-10-300, CA23-10-400, CA23-10-500, CA23-10-600, and CA23-10-700, respectively. Ultimately, the calcination-obtained high-purity modified melilite adsorbent was pulverized into fine powder for the subsequent adsorption experiments.

### 2.3. Adsorption Experiments

This study systematically investigated the fluoride adsorption performance of calcined-state mayenite (Ca_12_Al_14_O_33_) in water through batch adsorption experiments. All batch experiments were conducted in a thermostatic orbital shaker (200 rpm) using 100 mL of fluoride solution (100 mg L^−1^). The systematic parameter screening included adsorbent mass (0.2~2 g L^−1^) and solution pH (3~12). The adsorption kinetics, thermodynamics, and isotherms were investigated at different times (5~120 min), fluoride concentrations (100~800 ppm), and temperatures (25~45 °C) under an adsorbent dosing of 1 g L^−1^ and pH 7 ± 0.1. Post-adsorption suspensions were filtered through 0.45 μm membranes, with residual F^−^ concentrations determined by an ion meter (PXSJ-6F, Nanjing Everich Medicare Import & Export Co., Nanjing, China) equipped with a fluoride ion-selective electrode (PF-2-01, Shanghai Yidian Scientific Instrument Co., Shanghai, China), TISAB buffer was introduced prior to the measurements to eliminate ionic interference. Each trial included duplicate runs, with adsorption capacity (*qₑ*) and removal efficiency (*Rₑ*) calculated via Equations (1) and (2), respectively.

(1) $$q_{e}=\frac{\left(C_{0}-C_{t}\right)V}{m}$$

(2) $$R_{e}=\frac{\left(C_{0}-C_{t}\right)}{C_{0}}\times 100\%$$

where *C*_0_ and *C_t_* are the initial and post-adsorption fluorine concentrations (mg L^−1^), respectively; *V* is the solution volume (L); and m is the mass of adsorbent (g).

### 2.4. Characterization Tools

The pH value of the solution was measured by a PHS-3C pH meter (Shanghai Puchun Measure Instrument Co., Ltd., Shanghai, China), and the morphological characteristics and elemental distribution of the materials were observed by field-emission scanning electron microscope (SEM) model S-4800 (Hitachi, Tokyo, Japan) and transmission electron microscope (TEM) model JEM-2100F (JEOL Ltd., Tokyo, Japan) and X’Pert PRO X-ray diffractometer (XRD) (Panalytical, Almelo, The Netherlands). Structural–physical phase analyses of the materials were carried out by the BET and BJH methods, NoVA 1200e (Quantachrome Instruments, Inc., Boynton Beach, FL, USA) surface area, and porosity analyzer to determine the specific surface area, pore volume, and pore size distribution of the adsorbent and the Nicolet 6700-NXR Fourier-transfer infrared spectrometer (FT-IR) (Thermo Fisher Scientific Inc., Waltham, MA, USA) and EscaLab 250Xi X-ray photoelectron spectroscopy (XPS) (Thermo Fisher Scientific Inc., Waltham, MA, USA) to study the changes in the functional groups and the surface chemical properties of the materials.

## 3. Results and Discussion

### 3.1. Effects of Preparation Condition

#### 3.1.1. Ca/Al Molar Ratio

**Figure 1:**
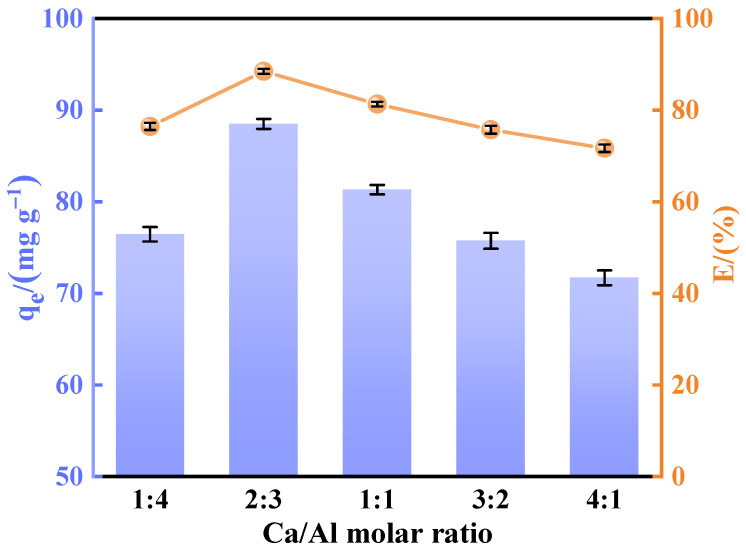
Effect of Ca/Al molar ratio on fluoride removal performance.

**Figure 2:**
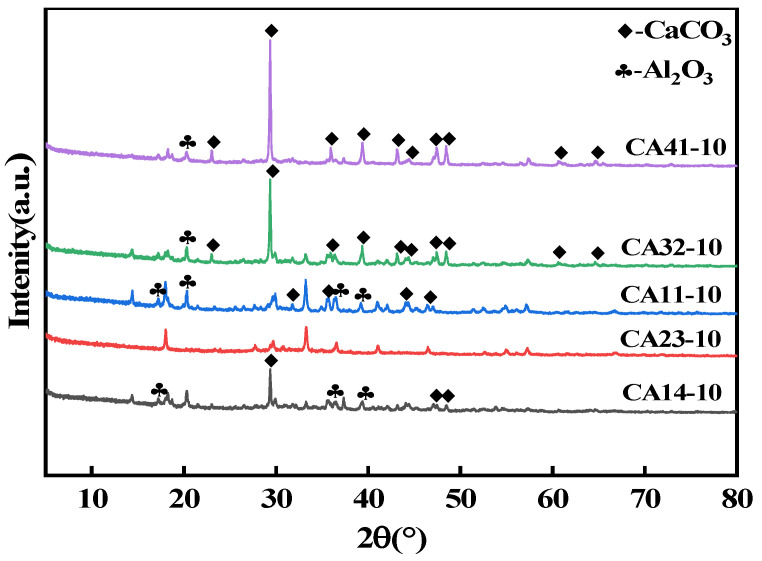
XRD spectra of CaAl-LDO synthesized at different Ca/Al molar ratios with pH 10 and calcination temperature of 600 °C.

To investigate the effect of composition on fluoride removal performance, adsorbents with varying Ca/Al molar ratios were synthesized using the co-precipitation method. A series of experiments were conducted to optimize the adsorbent by adjusting the Ca/Al molar ratio. As shown in Figure 1, the Ca/Al molar ratio significantly influences the fluoride removal capacity of the adsorbent. From the figure, it is evident that the adsorption capacity increases with the rise in Ca content and then decreases. At a Ca/Al molar ratio of 2:3, the fluoride removal performance remains high, likely due to the material’s surface properties and structural characteristics at this ratio. X-ray diffraction analysis (Figure 2) of the CaAl-LDO materials synthesized with different Ca/Al ratios revealed that the 2:3 molar ratio yielded materials with a superior crystallinity and well-defined crystalline structure, devoid of any apparent impurity phases. The characterization results suggest that excessive Ca^2+^ content promotes the formation of dense, calcium-rich structures dominated by CaCO_3_ phases, while excessive Al^3+^ leads to the development of fibrous alumina structures. Therefore, the optimization of the Ca/Al molar ratio is crucial for the improvement of the defluorination performance of the materials, and a reasonable Ca/Al ratio can not only enhance the structural stability of the adsorbent but also effectively improve its fluorine removal capability.

#### 3.1.2. pH of Precipitation

**Figure 3:**
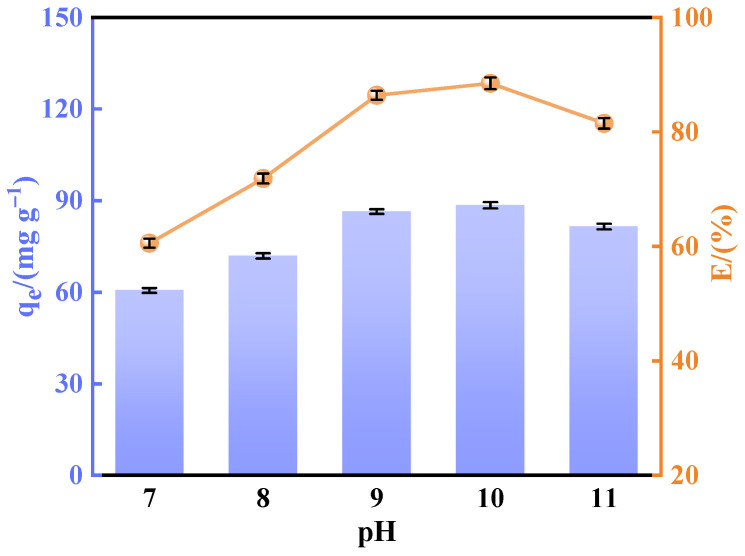
Effects of precipitation process pH value on performance of fluoride removal.

In the co-precipitation reaction of the mayenite (Ca_12_Al_14_O_33_) precursor, pH is one of the key factors in the regulation of the reaction process. Variations in pH directly influence the precipitation kinetics of metal ions, as well as the morphology, surface properties, and adsorption capacity of the final product. As shown in Figure 3, the defluoridation efficiency of the calcined calcium aluminate increased significantly within the pH range of 7–10, reaching a maximum value of 88.49% at pH 10. This trend indicates that moderately alkaline conditions facilitate the structural evolution of the mayenite phase (Ca_12_Al_14_O_33_), enhancing its fluoride adsorption capacity through improved hydroxyl group availability and an optimized surface charge distribution.

The pHpzc of the samples prepared at pH 7–11 were 6.64, 7.05, 7.39, 7.67, and 7.43. It was found that the optimal equilibrium of hydroxyl density and crystallinity on the surface of the material was achieved at pH 10 with a pHpzc of 7.67, which is close to the neutral range, allowing the material to maintain a moderately positive electrical charge in the fluoride-removing environment at pH 6–7. This property is able to enhance the adsorption of fluoride ions through electrostatic interaction, while avoiding a reduction in active sites due to overprotonation. However, at a low alkaline pH (7–10), calcium and aluminum ions were not sufficiently hydrolyzed, resulting in an incomplete formation of metal oxides and lack of effective adsorption sites on the surface of the material, which reduced the fluorine adsorption capacity. On the other hand, at too high a pH (>10), excess NaOH led to an increase in alkalinity, which in turn promoted secondary precipitation and the structural loosening of marvinite. In addition, excess OH^−^ ions may compete with fluoride ions for adsorption sites and inhibit the growth of crystals, which in turn negatively affects the adsorption performance [29,30,31].

These findings underscore the necessity of pH optimization for synthesizing high-efficiency fluoride adsorbents. Therefore, the optimization of the solution’s pH is the key to the preparation of highly efficient fluorine adsorbents, Both overly acidic and alkaline conditions degrade the adsorption capacity through distinct mechanisms.

#### 3.1.3. Calcination Temperature

**Figure 4:**
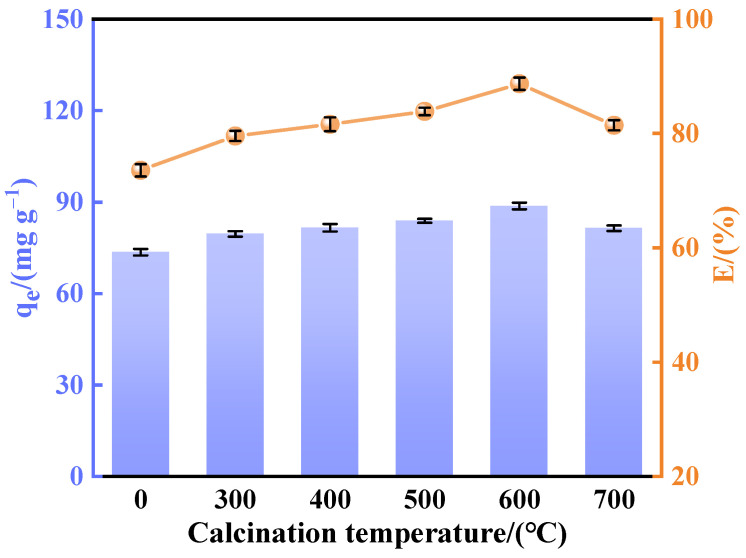
Effect of calcination temperature on performance of fluorine removal.

**Figure 5:**
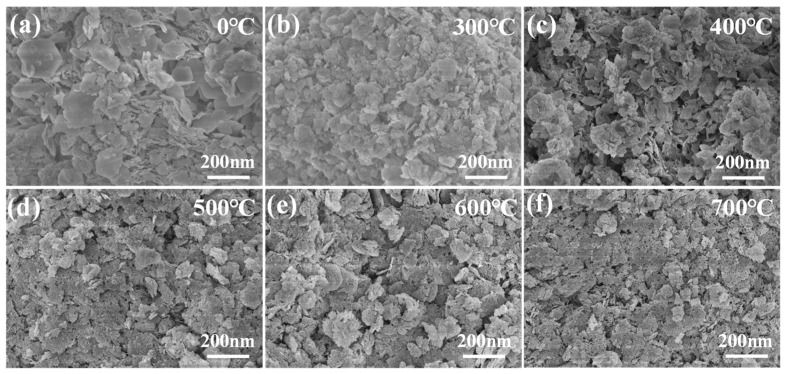
SEM morphology of Ca_12_Al_14_O_33_ at different calcination temperatures: 0 °C (**a**), 300 °C (**b**), 400 °C (**c**), 500 °C (**d**), 600 °C (**e**), 700 °C (**f**).

As shown in Figure 4, the calcination temperature has a significant effect on the adsorption properties of Ca_12_Al_14_O_33_, which is in agreement with the results of previous research [32,33]. Fluorine removal experiments were carried out by calcined CaAl-LDH obtained by the co-precipitation method at different temperatures (300~700 °C) for 2 h. The results showed that the fluorine removal performance of all the calcined samples was significantly enhanced compared with that of the untreated pristine CaAl-LDH, which suggests that the calcination treatment contributes to the enhancement of fluorine adsorption capacity. However, the fluorine removal performance increased initially and then decreased as the calcination temperature rose, indicating that adsorption capacity is closely linked to the adsorbent’s structure. The optimal fluorine removal effect was achieved at a specific temperature. SEM scans in Figure 5 show that below 500 °C, the material retains its layered structure. However, above 500 °C, metal hydroxide decomposition leads to the formation of pores on the surface, increasing the contact area with fluoride ions and enhancing adsorption. The optimal fluorine removal performance was observed at 600 °C. When the temperature exceeded 600 °C, particle agglomeration occurred, damaging the pore structure and transforming CaAl hydrotalcite into stable spinel. This transformation prevented the regeneration of the original hydrotalcite structure and caused the loss of its ‘memory effect’, leading to a decrease in adsorption performance. These results align with the theory of an optimal calcination temperature, which suggests that the best temperature balances the formation of mixed oxides with structural stability, optimizing the material’s pore structure and surface active sites [34].

### 3.2. Characteristics of Ca_12_Al_14_O_33_

**Figure 6:**
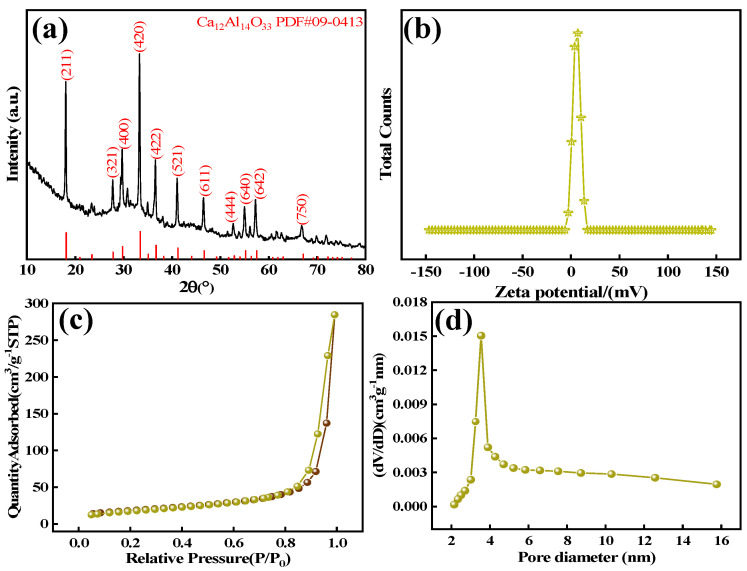
XRD (**a**), zeta potential curve (**b**), nitrogen adsorption–desorption isotherm (**c**), and pore size distribution curve (**d**) of Ca_12_Al_14_O_33_ adsorbent materials.

The crystal structure of the adsorbent material was confirmed by X-ray diffraction (XRD) patterns (Figure 6a), exhibiting characteristic Bragg reflections. The reflection peaks of Ca_12_Al_14_O_33_ synthesized by calcination at 2θ values of 18.13°, 27.82°, 29.78°, 33.41°, 36.70°, 41.21°, 46.66°, 55.22°, and 57.52° corresponded to the (2 1 1), (3 2 1), (4 0 0), (4 2 0), (4 2 2), (5 2 1), (6 1 1), (6 4 0), and (6 4 2) crystal planes, respectively, and the diffraction peaks were of a high intensity, and no impurity peaks were examined, which indicates that the product was of a high purity and complete crystallinity, and this suggests that the calcined-state mayenite (Ca_12_Al_14_O_33_)was successfully prepared.

Zeta potential is a key parameter to characterize the nature of the surface charge of adsorbents and their stability. As the zeta potential increases, the electrostatic repulsion between particles increases, and the dispersion and stability improve. Figure 6b shows that the zeta potential of Ca_12_Al_14_O_33_ is about 6.32 mV, and the surface is positively charged, indicating that the material has a good dispersion in an aqueous solution and resists particle aggregation. This positive charge enables the adsorbent to attract negatively charged fluoride ions (F^−^) through electrostatic forces, thereby enhancing its ability to efficiently adsorb fluoride ions.

The adsorption performance of adsorbent materials is strongly influenced by their specific surface area, pore size, and surface porosity. Analysis of the nitrogen adsorption–desorption isotherms and pore size distribution of the Ca_12_Al_14_O_33_ adsorbent (Figure 6c,d) reveals typical type IV isotherms with H3 hysteresis loops, indicating a mesoporous structure with slit-like pores. This structure arises from the formation of lamellar structures during the calcination process of calcium–aluminum bimetallic hydroxides due to water evaporation. The specific surface area, pore volume, and average pore diameter of Ca_12_Al_14_O_33_ were measured at 69.42 m^2^ g^−1^, 0.063 cm^3^ g^−1^, and 5.138 nm, respectively, confirming its mesoporous nature. The pore size, significantly larger than the average fluoride ion diameter (0.133 nm), allows the easy penetration of fluoride ions, thus enhancing its adsorption capacity [35]. In conclusion, the Ca_12_Al_14_O_33_ adsorbent’s high purity and porosity demonstrate its potential for diverse applications in adsorbent materials.

**Figure 7:**
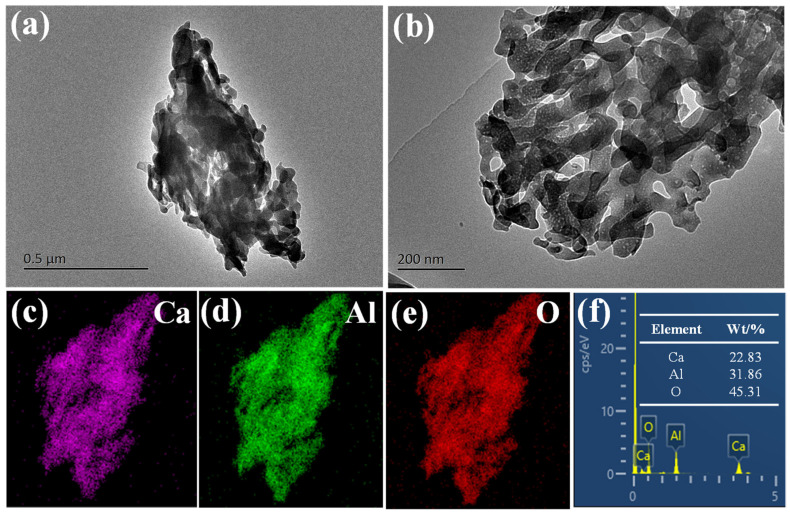
FETEM images (**a**,**b**), elemental mapping (**c**–**e**), and elemental content spectra (**f**) of Ca_12_Al_14_O_33_.

In addition, the microstructure of the Ca_12_Al_14_O_33_ adsorbent was examined in detail using transmission electron microscopy (TEM). The FETEM image in Figure 7a,b reveals a distinct hierarchical porous structure. Elemental distribution maps in Figure 7c–e show a uniform distribution of Ca, Al, and O elements, and the elemental content analysis in Figure 7f indicates the following percentages: Ca (22.83%), Al (31.86%), and O (45.31%). These elemental distributions are basically consistent with the theoretical calcium–aluminum molar ratio of 2:3, which further verifies the successful synthesis of Ca_12_Al_14_O_33_ adsorbent and the accuracy of its structure. The microstructural features indicate that the material has an excellent structural consistency and good elemental distribution, which provides an important guarantee for its stability and efficient adsorption performance during the adsorption process. In addition, the uniform elemental distribution helps to enhance the interaction between the Ca_12_Al_14_O_33_ adsorbent and the target substances, which further improves its effectiveness and reliability in practical applications.

### 3.3. Fluoride Adsorption Study

#### 3.3.1. Effect of Adsorbent Mass

**Figure 8:**
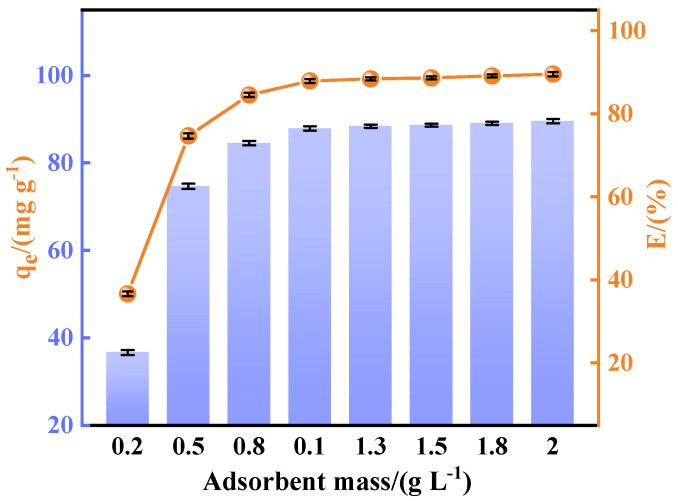
Effect of adsorbent mass on performance of fluoride removal.

Adsorbent mass is a key factor affecting the fluoride adsorption process. The adsorption capacity and removal rate of the Ca_12_Al_14_O_33_ adsorbent were evaluated in the adsorbent mass range of 0.2 g L^−1^~2 g L^−1^. The experimental conditions were a fluoride ion solution concentration of 100 mg L^−1^, stirring speed of 200 rpm, pH 7, and room temperature (25 °C). Figure 8 shows the adsorption capacity and removal rate at different adsorbent doses. The fluorine removal rate increased significantly with the increase in adsorbent mass when the adsorbent addition was in the range of 0.2~1 g L^−1^, driven by the proportional rise in available active sites for fluoride ion sequestration. Beyond 1.0 g L^−1^, however, the removal efficiency plateaued at 88.36%, with diminishing returns observed for further mass increments. This saturation behavior arises from particle agglomeration at excessive mass, which reduces the effective specific surface area and elongates the diffusion pathways for fluoride ions, ultimately compromising the adsorption kinetics [36]. Moreover, an elevated adsorbent loading intensifies solid–liquid separation challenges and escalates the operational costs, without commensurate performance gains. Consequently, a mass of 1.0 g L^−1^ was optimized to maximize the adsorbent’s utilization, while balancing removal efficiency, process economics, and post-treatment feasibility.

#### 3.3.2. Effect of pH Value

**Figure 9:**
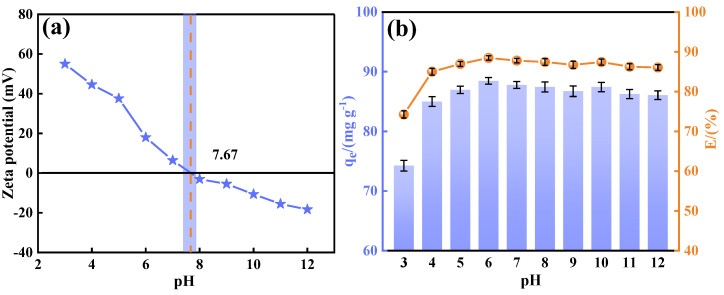
Zeta potential curves of Ca_12_Al_14_O_33_ (**a**) and the effect of the initial pH on fluorine adsorption capacity and removal efficiency (**b**).

The solution’s pH not only affects the chemical form of the adsorbent in solution, but it also influences its adsorption performance by changing the surface charge of the adsorbent and thus its adsorption performance. This study assessed the adsorption performance of Ca_12_Al_14_O_33_ on fluoride solutions with varying initial pH levels by examining the relationship between zeta potential and adsorption capacity. The experimental results are shown in Figure 9a,b.

As illustrated in Figure 9a, the point of zero charge (PZC) of the Ca_12_Al_14_O_33_ adsorbent was approximately 7.67. Zeta potential measurements indicated a gradual decrease in surface charge density as the pH increased from 2 to 12. The higher zeta potential values observed under acidic conditions correspond to enhanced colloidal stability and stronger positive surface charges, which favor electrostatic interactions.

Adsorption experiments performed at a constant fluoride concentration (100 mg L^−1^) showed pH-dependent behavior (Figure 9b). The highest fluoride removal efficiency (88.47%) was observed at pH 6. Fluoride adsorption increased gradually with a pH between 4 and 7, but dropped sharply when the pH was below 4. This behavior can be attributed to the fact that fluoride ions primarily exist as HF^−^ at a lower pH, while Ca_12_Al_14_O_33_ exhibits weak binding with HF^−^. Additionally, under acidic conditions, the partial dissolution of Ca_12_Al_14_O_33_ reduces the available adsorbent mass, which contributes to the significant decrease in fluoride removal efficiency as the pH increases; fluoride ions in water mainly exist in the form of F^−^, and the adsorbent is more likely to adsorb F^−^, resulting in an increase in fluorine adsorption. However, when the pH continued to increase above 6, the adsorption amount gradually decreased, which was mainly due to the increase in OH^−^ concentrations, resulting in deprotonation of the Ca_12_Al_14_O_33_ surface, and at the same time, the electrostatic repulsion between OH^−^ and F^−^ increased, and the competition for the adsorption active site became more obvious. When the pH was higher than the isoelectric point of the Ca_12_Al_14_O_33_ adsorbent (7.67), the surface of the material was negatively charged, which was unfavorable for the removal of fluoride ions. However, the adsorption of fluoride ions by Ca_12_Al_14_O_33_ remained high in the pH range of 4 to 12. Compared with other reported materials [37], Ca_12_Al_14_O_33_ shows a significant competitive advantage over a wide pH range, indicating its broad applicability as a fluoride adsorbent.

### 3.4. Adsorption Kinetic Analysis

**Figure 10:**
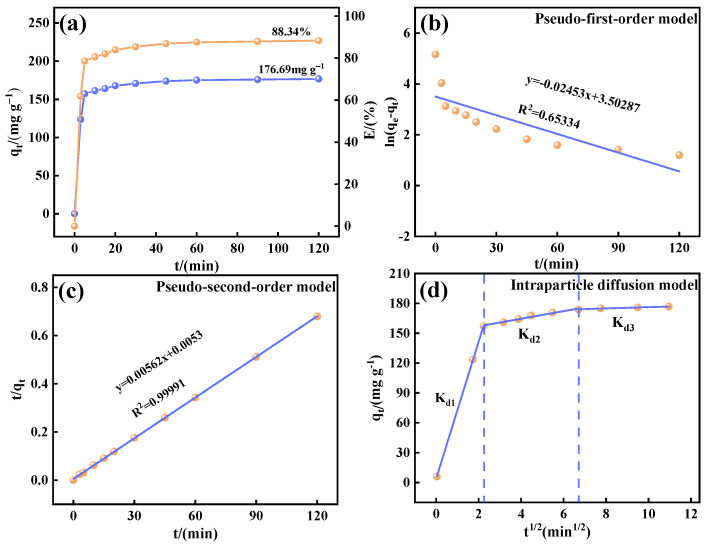
Variation patterns of adsorption of fluorine by Ca_12_Al_14_O_33_ with time (**a**), pseudo-first-order kinetic model (**b**), pseudo-second-order kinetic model (**c**), and intra-particle diffusion kinetic model (**d**).

The adsorption kinetics of Ca_12_Al_14_O_33_ for fluoride ions in wastewater were investigated. Figure 10a illustrates the kinetic curve of fluoride adsorption by Ca_12_Al_14_O_33_ nanomaterials, which can be divided into three stages. In the initial rapid phase (0~5 min), the adsorption rate was very high, with an about 78.6% removal within 5 min. This rapid uptake is attributed to the abundant surface sites on the Ca_12_Al_14_O_33_, which promoted a fast mass transfer driven by the concentration gradient, allowing efficient fluoride ion trapping. As time progressed, the adsorption entered a slower phase (5~60 min), during which the fluoride concentration decreased, and the active sites on the adsorbent surface became progressively occupied. This limited the available sites for further adsorption, leading to stronger repulsive forces between fluoride ions and the solution, thereby slowing the rate. After 60 min, the system reached equilibrium, with a fluoride adsorption capacity and removal efficiency of 176.69 mg g^−1^ and 88.34%, respectively. These results highlight the material’s rapid adsorption and excellent fluoride removal performance, making it ideal for water purification.

To further analyze the fluoride adsorption process by Ca_12_Al_14_O_33_, the widely accepted pseudo-first-order and pseudo-second-order kinetic models were applied to fit the experimental data. The corresponding linear equations are presented in Equations (3) and (4).

(3) $$\;\ln\left(q_{e}-q_{t}\right)=\ln q_{e}-K_{1}t\;$$

(4) $$\;\frac{t}{q_{t}}=\frac{1}{K_{2}q_{e}^{2}}+\frac{t}{q_{e}}$$

where *q_e_* and *q_t_* (mg g^−1^) represent the amounts of fluoride adsorbed by the adsorbent at equilibrium and at time t(s), respectively; and *K*_1_ (1/min) and *K*_2_ (g/(mg min)) are the rate constants for the pseudo-first-order and pseudo-second-order kinetic models, respectively.

The fitting results, presented in Figure 10b,c and Table 2, show that the quasi-second-order kinetic model has a higher correlation coefficient (0.9999) and a calculated adsorption capacity (177.936 mg g^−1^) closer to the actual value (176.69 mg g^−1^). This suggests that the fluoride adsorption process by Ca_12_Al_14_O_33_ follows the pseudo-second-order model, indicating that it is primarily governed by chemisorption [38].

To further explore the mass transfer mechanism and rate-limiting step of fluoride adsorption by Ca_12_Al_14_O_33_, the contact time data were analyzed using the intraparticle diffusion model, with the model’s linear equation presented in Equation (5).

(5) $$q_{t}=K_{\mathrm{di}}t^{0.5}+C_{i}$$

where *q_t_* (mg g^−1^) is the amount of fluoride adsorbed at time t(s), *k_di_* (mg/g min^0.5^) is the intraparticle diffusion rate constant, and *C* (mg g^−1^) represents the boundary layer constant.

Figure 10d shows the plots of qt versus t^1/2^ at different initial fluoride concentrations, with the corresponding K_d1_, K_d2_, and K_d3_ values listed in Table 3. The figure clearly demonstrates that the fitted curves exhibit multiple linear characteristics and do not pass through the origin, suggesting that intraparticle diffusion is not the sole rate-limiting step. Additionally, the kinetic parameters in Table 4 indicate that the diffusion rate constants for the three phases decrease in the order K_d1_ > K_d2_ > K_d3_, revealing that the adsorption process is influenced by multiple, synergistic steps: the initial rapid diffusion stage (K_d1_) may correspond to the boundary layer effect, and the subsequent slowing down of the K_d2_ and K_d3_ stages reflect intra-particle diffusion resistance and adsorption site saturation, respectively, which confirms the kinetic characteristics of the multi-mechanisms acting together [39,40].

### 3.5. Adsorption Activation Energy Studies

**Figure 11:**
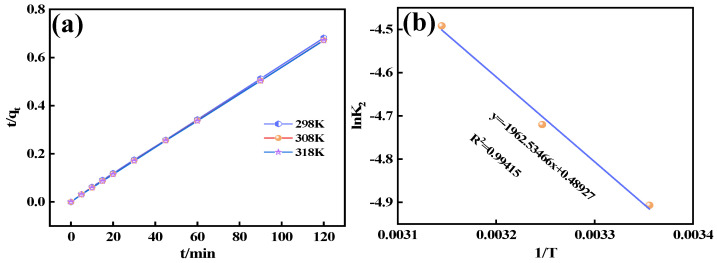
Pseudo-second-order kinetic model at different temperatures (**a**) and Arrhenius curve derived from the pseudo-second-order kinetic model (**b**).

Figure 11a displays the quasi-second-order kinetic curves at various temperatures, with the relevant parameters provided in Table 4. The results indicate that the reaction rate constant (K_2_) increases with temperature, suggesting that the fluorine adsorption rate by Ca_12_Al_14_O_33_ accelerates as the temperature rises, which implies that the adsorption process is endothermic. Figure 11b presents the plot of ln(K_2_) versus 1/T, and from this, the activation energy for fluorine adsorption was calculated to be 16.32 kJ mol^−1^. Based on the kinetic fitting results, it can be inferred that the adsorption process is primarily controlled by chemisorption. Typically, adsorption activation energy correlates with the cyclic adsorption performance of the adsorbent: a higher activation energy indicates more difficulty in desorbing the adsorbate. Therefore, Ca_12_Al_14_O_33_ not only exhibits an excellent adsorption capacity for fluorine but also demonstrates favorable desorption characteristics.

### 3.6. Adsorption Isotherm Studies

**Figure 12:**
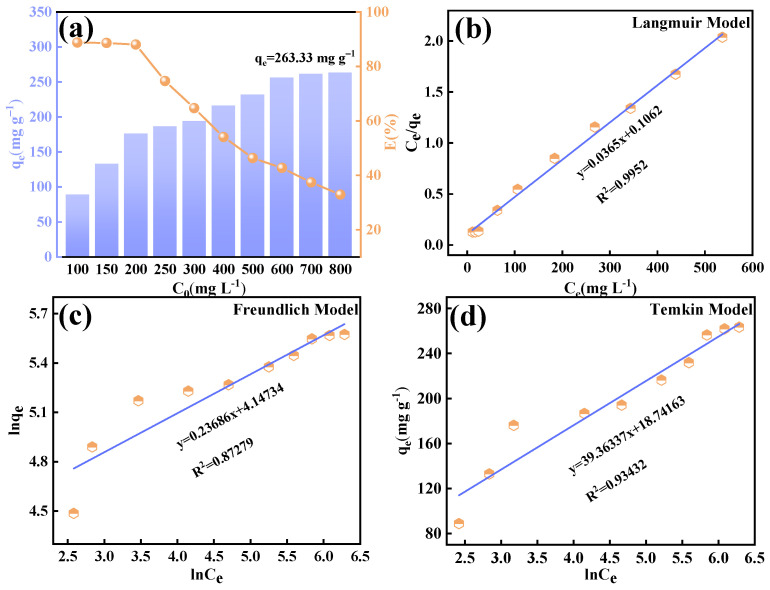
Adsorption isotherm curves of Ca_12_Al_14_O_33_ at different fluorine concentrations (**a**) and Langmuir model (**b**), Freundlich model (**c**), and Temkin model (**d**).

The adsorption isotherm investigates the relationship between the adsorbed amount of adsorbent and the equilibrium concentration of fluoride ions in the solution when the solid adsorbent reaches dynamic equilibrium with the fluoride-containing solution at the solid-liquid interface. Figure 12a shows the adsorption capacity and removal rate of Ca_12_Al_14_O_33_ at different fluoride concentrations of 100~800 mg L^−1^. The results show that the adsorption capacity increases with the increase in the initial concentration of fluoride until it reaches an equilibrium adsorption capacity of 263.33 mg g^−1^, whereas the removal rate shows a decreasing trend from 88.78% to 32.92%. This was attributed to the fact that at lower fluorine concentrations, the surface active sites of the adsorbent were abundant, and the fluoride ions were rapidly bound through ligand bonds, which drove a rapid increase in the adsorption amount; however, the adsorption active sites on the Ca_12_Al_14_O_33_ tended to saturate with the increase in the concentration of the fluoride solution, and when the concentration of fluoride solution reached a certain level, the concentration gradient-driven mass transfer was restricted, resulting in the adsorption amount entering a plateau period. Further increases in fluoride concentration lead to a saturation of the adsorption capacity, which in turn reduces the removal rate at higher concentrations.

To further investigate the adsorption behavior and mechanism of fluoride ions on Ca_12_Al_14_O_33_ nanoparticles, experimental data for fluoride ions at various concentrations were fitted to the Langmuir, Freundlich, and Temkin isotherm models. The fitting curves are shown in Figure 12b–d, with the corresponding fitting parameters listed in Table 5. The results indicated that the Langmuir model, with a correlation coefficient (R^2^) of 0.9952 and a theoretical saturated adsorption capacity of 273.97 mg g^−1^, provided a closer fit to the actual adsorption compared to the other models. This suggests that the fluoride ion adsorption on Ca_12_Al_14_O_33_ nanoparticles follows a monolayer adsorption process [41].

### 3.7. Adsorption Thermodynamic Studies

**Figure 13:**
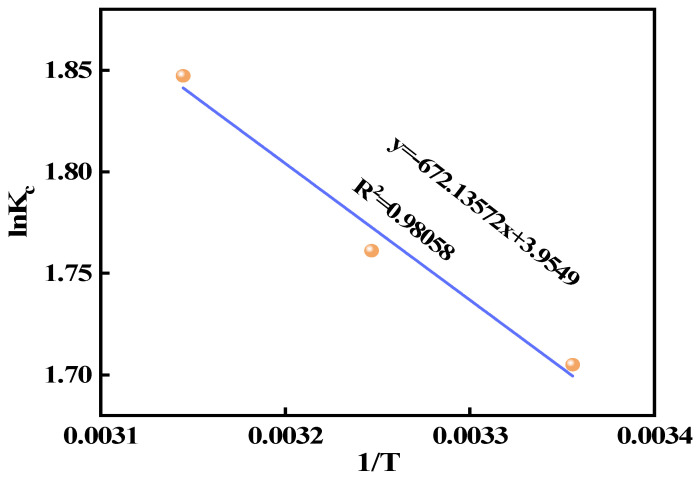
Thermodynamic modeling analysis.

Figure 13 shows the thermodynamic diagram for the adsorption of fluoride ions by Ca_12_Al_14_O_33_ nanoparticles, with the corresponding thermodynamic parameters provided in Table 6. The results reveal that the ΔG values are negative at all temperatures, with the absolute value increasing as the temperature rises. This suggests that the adsorption process is spontaneous and thermodynamically driven, and that higher temperatures favor the reaction. A positive enthalpy change (ΔH = 5.588 kJ mol^−1^) confirms the endothermic nature of the adsorption mechanism. Concurrently, the positive entropy variation (ΔS = 32.881 J mol^−1^ K^−1^) reveals increased system disorder during fluorination, primarily attributed to interfacial interactions at the solid–liquid boundary. These collective findings establish that the defluorination process mediated by Ca₁₂Al₁₄O₃₃ nanoparticles operates through a spontaneous, entropy-driven mechanism with inherent endothermic characteristics [42].

### 3.8. Adsorption Mechanism

In order to further explore the adsorption mechanism of Ca_12_Al_14_O_33_ on fluorine in water, the Ca_12_Al_14_O_33_ adsorbent was analyzed using FT-IR and XPS.

**Figure 14:**
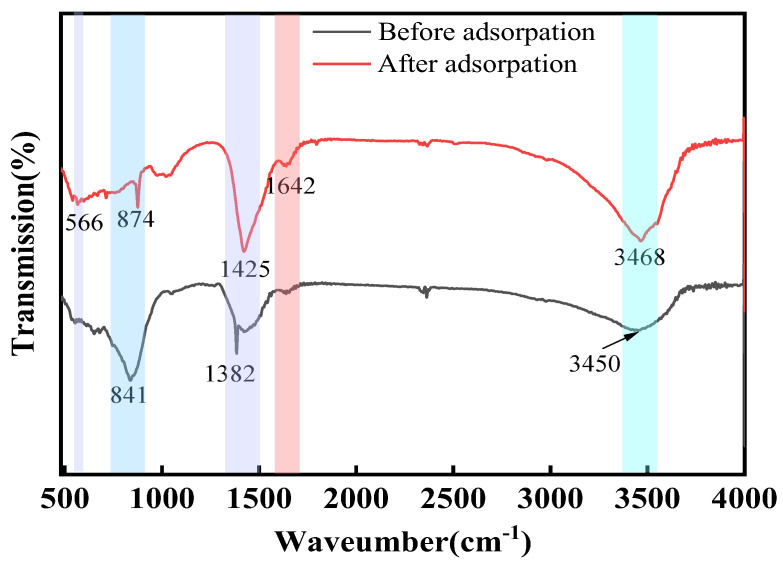
FT-IR spectra of Ca_12_Al_14_O_33_ before and after fluorine adsorption.

Figure 14 shows the FT-IR pattern of Ca_12_Al_14_O_33_ before and after fluorine adsorption. The band at 3450 cm^−1^ is attributed to the stretching vibration of the -OH group in Ca_12_Al_14_O_33_. After adsorption, the -OH stretching vibration shifted from 3450 cm^−1^ to 3468 cm^−1^, indicating that the -OH in Ca_12_Al_14_O_33_ interacted with the fluoride ions in solution [43]. The absorption peaks near 1425 cm^−1^ corresponded to M-O and M-OH (M = Ca, Al) [23]. After the adsorption of fluoride, the absorption peak at 1382 cm^−1^ disappeared, suggesting that fluoride may have been adsorbed on the corresponding hydroxyl sites. Since the dimensions of both the -OH and F^−^ groups are almost the same, they can be exchanged for each other, allowing F^−^ to bind to the adsorbent. In the low-frequency range of 500~900 cm^−1^, the absorption peaks were mainly caused by Ca/Al-O vibrations. The peak value of the absorption peak of Ca_12_Al_14_O_33_ near 841 cm^−1^ became smaller after adsorption, indicating that the metal–oxygen bonding and F^−^ were intercomplexed. In addition, a new adsorption peak of Ca_12_Al_14_O_33_ at 566 cm^−1^ after adsorption corresponded to the formation of metal–fluoride complexes [44].

**Figure 15:**
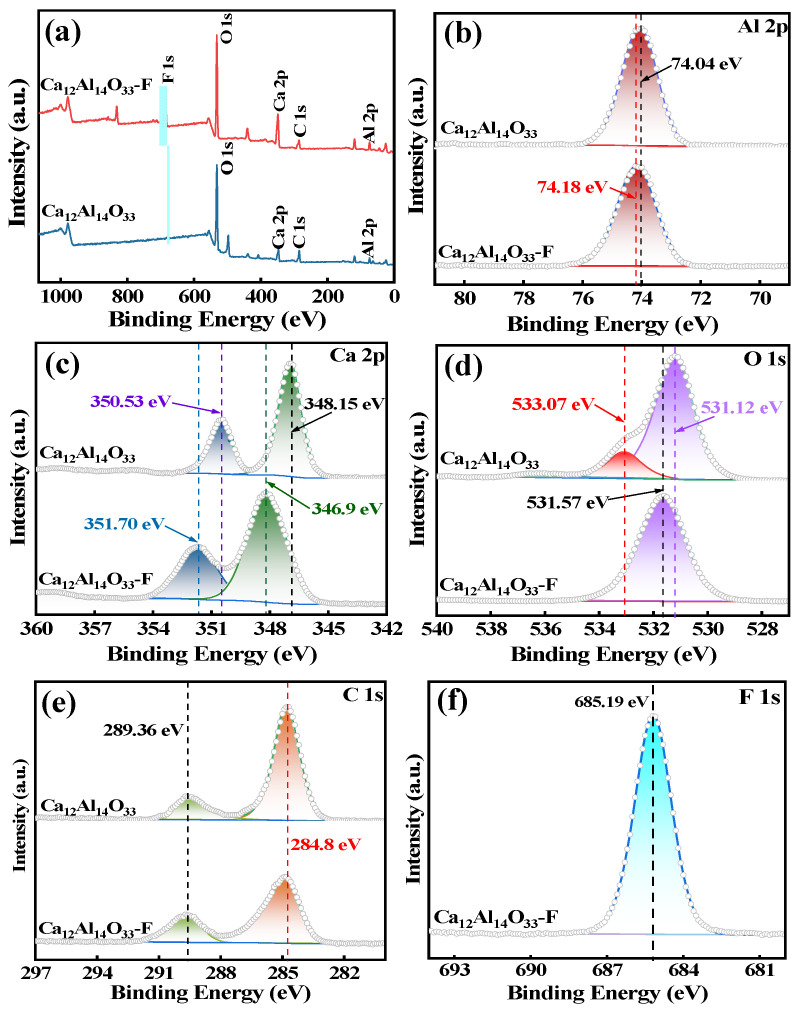
XPS spectra of Ca_12_Al_14_O_33_ before and after fluorine adsorption (**a**) and Al 2p (**b**), Ca 2p (**c**), O 1s (**d**), C 1s (**e**), and after fluorine adsorption F 1s (**f**) high-resolution XPS spectra.

The chemical composition and chemical state of the adsorbent before and after fluoride treatment were investigated using X-ray photoelectron spectroscopy (XPS), and the occurrence of Al, Ca, O, and F in the samples is shown in Figure 15a. The binding energy of Al 2p changed from 74.04 eV to 74.15 eV after fluoride adsorption (Figure 15b). For the Ca2p region, the peaks at 348.15 eV and 350.53 eV corresponded to Ca 2p3/2 and Ca 2p1/2, respectively [45], and the binding energies changed to 346.9 eV and 351.7 eV, respectively, after adsorption, and the separation of Ca2p peaks was observed (Figure 15c). Taken together, this indicates that fluoride undergoes strong complexation with the metal ions Ca/Al. The high-resolution O 1s spectra (Figure 15d) can be corresponded to Al-O-Al (531.12 eV) and Al-OH (533.07 eV), suggesting that the interlayer -OH of Ca_12_Al_14_O_33_ is involved in the reaction [29]. The C1 s peaks after adsorption in Figure 15e are C-C (284.8 eV) and C=O (289.36 eV), respectively. Importantly, as shown in Figure 15f, the adsorbed sample showed a new F 1s peak at 685.19 eV, confirming that the elemental fluorine was effectively captured by Ca_12_Al_14_O_33_.

In summary, the results of the FT-IR and XPS analyses showed that fluorine was effectively captured on the surface of the adsorbent by the ion exchange of fluoride with the -OH group on the surface of the Ca_12_Al_14_O_33_, as well as by the coordination of fluorine with the metal ion Ca/Al.

### 3.9. Regeneration and Recycling

The cyclic regeneration capacity of adsorbent is a key index to judge its performance. In this study, the regeneration capacity of the Ca_12_Al_14_O_33_ adsorbent was systematically investigated through a strategy combining ion exchange and calcination. Specifically, the Ca_12_Al_14_O_33_ material after saturated fluoride adsorption was dispersed in 0.5 M Na_2_CO_3_ solution and shaken continuously for 12 h at 25 °C on a constant-temperature shaker to induce ion exchange between F^−^ and CO_3_^2−^ in the layers. Subsequently, the samples were calcined at 500 °C for 2 h to completely remove the CO_3_^2−^ embedded in the interlayer, thus restoring the active sites of the adsorbent and achieving adsorbent regeneration [44].

**Figure 16:**
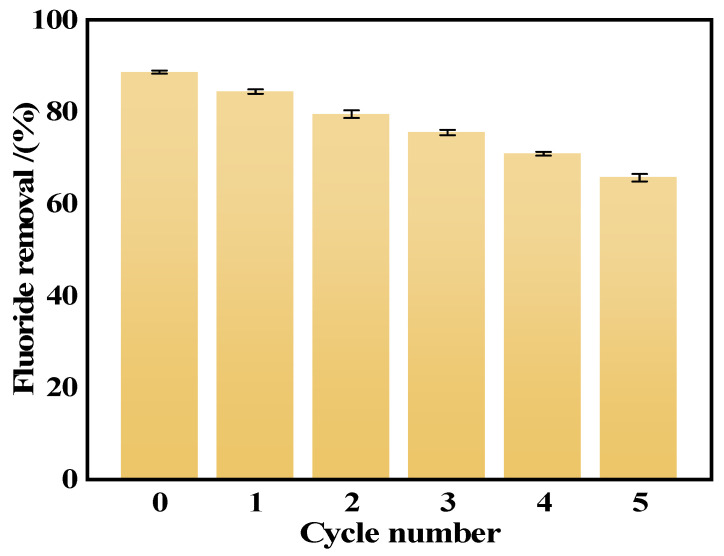
Cyclic adsorption properties of Ca_12_Al_14_O_33_.

Figure 16 shows that after five adsorption–desorption cycles, the removal of fluorine by the Ca_12_Al_14_O_33_ adsorbent decreased from the initial 88.74% to 65.92%, where the decay rate of the efficiency of each cycle was 3.8% on average. The incomplete desorption of fluorine, as well as pore clogging, were the main reasons for the decrease in the number of adsorbed active sites, as evidenced by the incomplete desorption of some fluoride ions during desorption and the possibility that the pore structure of the adsorbent was partially clogged by impurities or fluoride ion residues in the desorbent solution. Nevertheless, the Ca_12_Al_14_O_33_ adsorbent still showed a high recovery performance, which indicated that it was able to effectively recover its structure and adsorption capacity after the washing treatment of the desorption solution, and it demonstrated a strong stability and regeneration ability.

**Figure 17:**
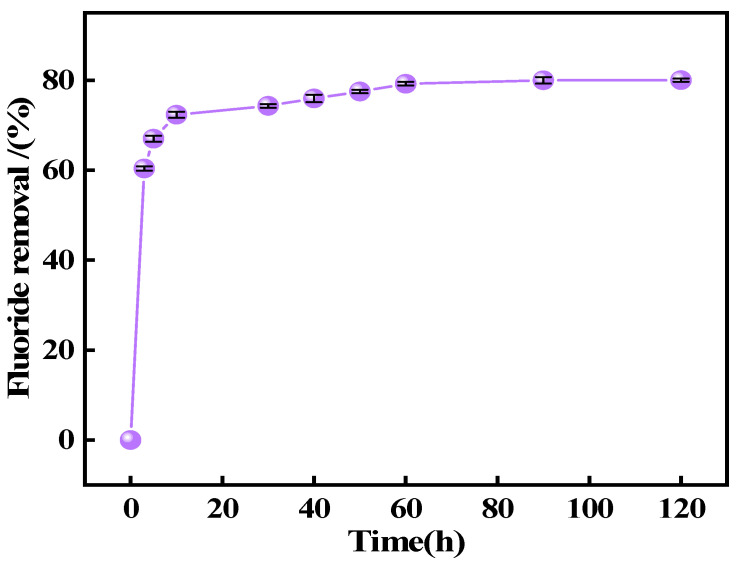
Removal of fluoride ions by Ca_12_Al_14_O_33_ in real fluoride smelting wastewater (initial fluoride ion concentration: 218.29 mg L^−1^, adsorbent dosage: 1.0 g L^−1^, adsorption time: 2.0 h).

The Ca_12_Al_14_O_33_ adsorbent developed in this study demonstrated excellent performance in a wastewater treatment system. We used the adsorbent to evaluate the removal rate of actual fluorinated wastewater (218.29 mg L^−1^) discharged from a copper smelting production process. The results showed that the adsorbent achieved a removal rate of 72.34% within 10 min, and this further increased to 80.03% within 2 h (Figure 17), with a performance similar to that in the simulated wastewater (Figure 9a).

### 3.10. Comparison of Ca_12_Al_14_O_33_ with Other Adsorbents

The comparative data in Table 7 indicate that the Ca_12_Al_14_O_33_ adsorbent synthesized in this study demonstrates a significantly stronger adsorption capacity for fluoride ions compared to a range of existing adsorbents. This material effectively removes fluoride ions in water treatment applications and shows great potential for practical use, offering an efficient solution to the problem of fluoride contamination.

## 4. Conclusions

In this study, we successfully synthesized a highly efficient fluoride ion adsorbent, calcined-state mayenite (Ca_12_Al_14_O_33_), by optimizing the co-precipitation–calcination process. Under optimal synthesis conditions (calcium/aluminum molar ratio of 2:3, pH 10, calcination temperature of 600 °C), the material exhibited a high specific surface area (69.42 m^2^ g^−1^), an ordered mesoporous structure (pore size of 5.138 nm), and abundant surface hydroxyl groups. The saturated adsorption capacity of the Ca_12_Al_14_O_33_ for fluoride ions was 263.33 mg g^−1^, significantly surpassing that of most comparable adsorbents. The adsorption kinetics followed a quasi-second-order model, and the Langmuir adsorption isotherm best described the process. Fluoride removal occurred mainly via ion exchange between the metal ions and surface hydroxyl groups. Thermodynamic analysis indicated that the adsorption process is spontaneous, endothermic, and entropy-increasing, making it suitable for practical applications. After five adsorption–desorption cycles, the material retained 65.92% of its initial adsorption capacity, demonstrating excellent structural stability and regeneration potential. Notably, in real fluorine-containing smelting wastewater, the material effectively removed fluoride ions. Compared to traditional adsorbents, Ca_12_Al_14_O_33_ offers significant advantages in adsorption capacity, pH adaptability, and regeneration performance, making it an efficient and cost-effective solution for wastewater treatment.

## Figures and Tables

**Table 1:** Composition of actual fluorinated wastewater from copper smelters.

Parameters	pH	F^−^	Cl^−^	SO_4_^2−^	As	Cu	Pb	Zn	Fe
Concentration (mg L^−1^)	4.7	218.29	59.86	50.33	73.34	15.98	7.89	53.97	8.36

**Table 2:** Kinetic parameters of fluorine adsorption by Ca_12_Al_14_O_33_.

Qe-Exp (mg g^−1^)	176.69		
Pseudo-first-order	qe-cal (mg g^−1^)	K_1_ (min^−1^)	R^2^
	27.85	0.02453	0.65334
Pseudo-second-order	qe-cal (mg g^−1^)	K_2_ (min^−1^)	R^2^
	177.936	0.00562	0.99991

**Table 3:** Parameters of the internal diffusion model.

K_d1_ (mg g^−1^ min^−1/2^)	C_1_	R_1_^2^
68.7431	3.9613	0.9999
K_d2_ (mg g^−1^ min^−1/2^)	C_2_	R_2_^2^
3.803	149.3697	0.9793
K_d3_ (mg g^−1^ min^−1/2^)	C_3_	R_3_^2^
0.6388	169.7996	0.9548

**Table 4:** Relevant parameters of quasi-second-order kinetic model at different temperatures.

Temperature/(K)	The Fitted Equations	K_2_/(min^−1^)	R^2^
298	y = 0.00564x + 0.0043	0.007398	0.9999
308	y = 0.00557x + 0.00348	0.008915	0.9999
318	y = 0.00557x + 0.00277	0.0112	0.9999

**Table 5:** Isothermal adsorption model parameters for fluorine adsorption by Ca_12_Al_14_O_33_.

Langmuir model	q_m_ (mg g^−1^)	K_L_	R^2^
	273.9726	0.3437	0.9952
Freundlich model	K_F_ (mg^1−(1/n)^L^1/n^g^−1^)	n	R^2^
	63.26549	0.66428	0.9811
Temkinm model	K_T_ (L mg^−1^)	b	R^2^
	0.34637	62.9411	0.97734

**Table 6:** Thermodynamic parameters of fluorine adsorption by Ca_12_Al_14_O_33_ at different temperatures.

Temperature/(K)	ΔG (kJ mol^−1^)	ΔH (kJ mol^−1^)	ΔS (J mol^−1^ K^−1^)
298	−4.2242	5.588	32.881
308	−4.5097		
318	−4.8838		

**Table 7:** Comparison of adsorption of F^−^ by Ca_12_Al_14_O_33_ with other previously reported adsorbents.

Adsorbents	Solution pH Value	*q_e_* (mg g^–1^)	References
Ce-Fe bimetal oxides	6.8	60.97	[46]
Mg/Al layered double hydroxides	6.0	27.03	[32]
Ce-La-MOFs	3	138.64	[47]
Fe-La composite	3.8–7.1	27.52	[48]
Ce-Al bimetallic oxides	7.0	146.73	[49]
Ca-Fe metal oxide	7.0	160.66	[27]
La–Zr composite	3–9	57.23	[50]
MMA	7.0	63.05	[51]
CCMZ	4–11	144.05	[37]
CZMA	4–12	84.24	[52]
Fe-Al-La composite	6.8	74.07	[53]
Ca_12_O_14_O_33_	4–12	263.33	This work

## Data Availability

The original contributions presented in the study are included in the article; further inquiries can be directed to the corresponding author.
